# Practices During Intubation in COVID-19 Intensive Care Units in India: A Cross-Sectional Questionnaire-Based Survey

**DOI:** 10.7759/cureus.34424

**Published:** 2023-01-31

**Authors:** Chinmaya K Panda, Subrata K Singha, Habib Md R Karim, Meghana Mishra

**Affiliations:** 1 Anesthesiology, Critical Care, and Pain Medicine, All India Institute of Medical Sciences, Raipur, IND; 2 Anesthesiology and Critical Care, DKS Super Specialty Hospital, Raipur, IND

**Keywords:** sars-cov-2, safety, healthcare workers, endotracheal intubation, covid-19, airway management, aerosol-generating procedures

## Abstract

Background

Healthcare workers are committed to learning from each other’s experience to safely optimize patient management of COVID-19. Acute hypoxemic failure is common in COVID-19 patients, and nearly 3.2% may require intubation. Intubation is an aerosol-generating procedure (AGP) that might predispose the performer to COVID-19 infection. This survey was intended to evaluate the practices during tracheal intubation in COVID-19 intensive care units (ICUs) and analyze them against the recommendations of the All India Difficult Airway Association (AIDAA) for safe practice.

Methodology

It was a web-based, multicentric cross-sectional survey. The choices in the questions were based on guidelines for airway management in COVID-19. Survey questions were divided into two parts - the first part consisted of demographics and general information, and the second part focused on safe intubation practices.

Results

A total of 230 responses were obtained from physicians all over India, presuming their active involvement in COVID-19 cases, of which 226 responses were taken into account. Two-thirds of responders did not receive any training before ICU posting. The Indian Council of Medical Research (ICMR) guideline was followed by 89% of responders for personal protective equipment use. Intubation in COVID-19 patients was predominantly conducted by a senior anesthesiologist/intensivist in the team and a senior resident (37.2%). Rapid sequence intubation (RSI) and modified RSI were preferable among the responder’s hospitals (46.5% vs. 33.6%). In most centers, responders used direct laryngoscope for intubation (62.8%), whereas video laryngoscope was used by 34%. Most responders confirmed the endotracheal tube (ETT) position by visual inspection (66.3%) over end-tidal carbon dioxide (EtCO2) concentration tracing (53.9%).

Conclusions

Safe intubation practices were followed in most of the centers across India. However, teaching and training, preoxygenation methods, alternative ventilation strategies, and confirmation of intubation pertinent to COVID-19 airway management need more attention.

## Introduction

Healthcare workers are committed to learning from each other's experience to safely optimize the management of severe acute respiratory syndrome coronavirus-2 (SARS-CoV-2) causing COVID-19. According to the World Health Organization, what initially started in Wuhan province, China, in December 2019, continued to escalate and has affected populations worldwide. Meng et al. reported that around 3.2% of patients required intubation at some point in the disease process [1]. Contact tracing of the COVID-19 cases indicates a human-to-human transmission of the disease, and symptomatic and asymptomatic SARS-CoV-2-infected patients can transmit the disease. Further evaluation of the mode of transmission of the virus indicated that SARS-CoV-2 could get transmitted through aerosol [2]. Airway management often requires interventions like preoxygenation, suctioning, and direct laryngoscopy. As preoxygenation is done in awake or slightly sedated patients, coughing during the procedure can generate an aerosol. Aerosol generation is also well-known during suctioning and laryngoscopy [3], especially when the patient is not paralyzed.

Further, direct laryngoscopy often necessitates a closer direct look into glottic openings, which brings the performer's face near the patient mouth and nose. Therefore, airway management is a potential intervention that can predispose the performer to getting infected, and measures to prevent it are necessary. As physicians/intensivists (INs)/anesthesiologists caring for moderate-to-severe COVID-19 patients frequently required to perform intubation, a study on the practices during the intubation procedure might help us understand the deficits and formulate plans. We have conducted an online survey to know the practices during intubation and analyze them against the guidelines provided by the All India Difficult Airway Association (AIDAA), emphasizing safe practices [4].

## Materials and methods

This web-based, cross-sectional survey was conducted over six weeks (August-September 2020). Thirty questions were sent to the physicians who indulged in the care of COVID-19 patients. At the time of conducting the survey, our institutional research rules for an online questionnaire-based survey where participants are not patients under treatment or follow-up, and prior approval was not regarded as mandatory. Further, as per the clinical trial registry of India, such studies do not need registration in the trial registry. However, online consent was sought and was considered as implied if the participant agreed to proceed with the survey.

Study design

The study was conceptualized and designed by a team of physicians working in the intensive care unit (ICU) designated for COVID-19 patients on a web-based platform (Google Forms, Mountain View, CA, USA). Questions were pertinent to the responder's demographics, practices, and measures for making aerosol-generating procedures (AGPs) safe during airway management. The questionnaire was based on a literature review relevant to intubation in COVID-19 ICUs. It was validated by five airway experts actively caring for COVID-19 patients. A few modifications were made after careful analysis by the research team. A pilot run of the questionnaire was done by sending the Google form/web-based link to 10 doctors to know the responder's interpretation. After evaluating the responses, appropriate changes were made to the options for various questions.

Thirty questions were segregated into two main parts - the first part was about the general information of the questionnaire, and the second part consisted of demographics and intubation practices in COVID-19. All the questions about airway management were of type multiple-choice and had both single-answer and multiple-answer options as required. Further, one question was kept open-ended. The questions with multiple feasible answers were marked and noted in the description. The nonprobability sampling technique of the snowball method was applied, and the online link to the questionnaire was sent to the probable responders through email and WhatsApp with a request to even forward the link to a known anesthesiologist/IN/physician through WhatsApp. Emails were collected from the various publicly available national institutes and academic society domains. A responder was allowed to take the survey only once, thus prohibiting multiple attempts. It was made compulsory to respond to all questions to enable the online submission of forms.

Statistical analysis

This study was designed to assess the compliance of intensivists to the guidelines provided by the AIDAA for airway management during the COVID-19 pandemic [4]. This is the first survey in COVID-19 on intubation practice; hence, determining the sample size was not possible. The data collected on the web-based platform (Google Sheets) was later converted into Microsoft Excel. Categorical variables were expressed as frequency (percentage). A chi-square test was applied to correlate the responses with safe practices for intubation during COVID-19.

## Results

A total of 230 responses were obtained from physicians all over India, presuming their active involvement in airway management in COVID-19 cases. Four responses were discarded as they did not reach the planned time frame. The result was analyzed based on 226 responses. Twelve responders whose hospitals were not admitting COVID-19 patients got deputed to different setups for managing COVID-19 cases in ICUs.

Sixty percent of responders were from tertiary healthcare centers/institutes of national importance (TH/INI), working in designated COVID ICUs and operation theaters (OTs). The responders from TH/INI were mostly having >10 years (34.3%) or one to five years (33.5%) of experience. The distribution of responders is presented in Table 1.

**Table 1:** Workspace and experience characteristics of the participants. CH, corporate hospitals; DC-ICU, COVID-19 intensive care unit; EMS, emergency medical services; OT, operation theaters; PGC, peripheral government COVID-19 centers; SGH, state government hospital; TH/INI, tertiary care hospital/institute of national importance

Particulars	Options	*n* (%)
Level of healthcare setup	PGC	4 (1.8)
	SGH	41 (18.1)
	CH	44 (19.5)
	TH/INI	137 (60.6)
Place of work	DC-ICU	148 (65.5)
	EMS	65 (28.8)
	OT	10 (4.4)
	Others	3 (1.3)
Hospital admitting COVID-19 cases	Yes	214 (94.7)
	No	12 (5.3)
Experience in intubation (years)	<1	16 (7.1)
	1-5	79 (35.0)
	5-10	52 (23.0)
	>10	79 (35.0)

Teaching, training, and preparedness

A total of 142 participants (62.8%) had not received any training for intubation during COVID-19, and it was grossly inadequate across all healthcare setups. Most participants (87.2%) did not use any scoring system for intubation. Among the healthcare setups, the scoring system adapted by CH was more (22.7%), but it was statistically insignificant (*P *> 0.05). Most responders (89.4%) followed the Indian Council of Medical Research (ICMR) guidelines for donning and doffing. Most healthcare setups did not have a negative pressure procedure room (83.6%) for intubation. Clamping of the endotracheal tube (ETT) before intubation was primarily practiced in SGH (51.2%), followed by TH/INI and CH (43.1% vs. 18.2%), which was statistically significant (*P *< 0.05). Most setups use heat and moisture exchangers and filters (HMEFs; 94.7%). Prone ventilation was practiced before intubation by 45.1% of responders. Postintubation decontamination of the room was done by 58.5% (SGH), followed by CH and TH/INI (52.3% vs. 35.05%), which was statistically significant (*P *< 0.05). All responses are shown in Table 2.

**Table 2:** Safe intubation practices among healthcare setups and their comparison. CH, corporate hospital; PGC, peripheral government COVID-19 centers; SGH, state government hospital; TH/INI, tertiary care hospital/institute of national importance; ICMR, Indian Council of Medical Research; ETT, endotracheal tube

Questionnaire	Total responses, *n* (%)			Healthcare setup	Individual responses, *n* (%)			*P*-value
	Yes	No	Not always		Yes	No	Not always	
Did you receive any formal training for intubation in a COVID-19 patient?	84 (37.2)	142 (62.8)	0	TH/INI	52 (38)	85 (62)	NA	0.17
				SGH	19 (46.3)	22 (53.7)	NA	
				CH	13 (29.5)	31 (70.5)	NA	
				PGC	0 (0)	4 (100)	NA	
Is donning and doffing done as per the ICMR guidelines?	202 (89.4)	24 (10.6)	0	TH/INI	124 (90.5)	13 (9.5)	NA	0.71
				SGH	35 (85.4)	6 (14.6)	NA	
				CH	39 (88.6)	5 (11.4)	NA	
				PGC	4 (100)	0 (0)	NA	
Do you use any scoring system for intubation?	29 (12.8)	197 (87.2)	0	TH/INI	15 (10.9)	122 (89.1)	NA	0.15
				SGH	4 (9.8)	37 (90.2)	NA	
				CH	10 (22.7)	34 (77.3)	NA	
				PGC	0 (0)	4 (100)	NA	
Is prone position ventilation being practiced before considering intubation in your setup?	102 (45.1)	73 (32.3)	51 (22.6)	TH/INI	56 (40.9)	51 (37.2)	30 (21.9)	0.27
				SGH	22 (53.7)	12 (29.3)	7 (17.1)	
				CH	23 (52.3)	9 (20.5)	12 (27.3)	
				PGC	1 (25)	1 (25)	2 (50.0)	
Do you have a negative pressure procedure room for intubation in your setup?	37 (16.4)	189 (83.6)	0	TH/INI	22 (16.1)	115 (83.9)	NA	0.45
				SGH	5 (12.2)	36 (87.8)	NA	
				CH	10 (22.7)	34 (77.3)	NA	
				PGC	0 (0)	4 (100)	NA	
Do you use any antifogging method for increased visibility during intubation in COVID-19 cases?	19 (8.4)	207 (91.6)	0	TH/INI	10 (7.3)	127 (92.7)	NA	0.21
				SGH	2 (4.9)	39 (95.1)	NA	
				CH	7 (15.9)	37 (84.1)	NA	
				PGC	0 (0)	4 (100)	NA	
Do you clamp ETT before intubation?	88 (38.9)	138 (61.1)	0	TH/INI	59 (43.1)	78 (56.9)	NA	0.05
				SGH	21 (51.2)	20 (48.8)	NA	
				CH	8 (18.2)	36 (81.8)	NA	
				PGC	0 (0)	4 (100)	NA	
Do you use a heat moisture exchanger and filter (HMEF) between ETT and the ventilator circuit?	214 (94.7)	12 (5.3)	0	TH/INI	130 (94.9)	7 (5.1)	NA	0.44
				SGH	37 (90.2)	4 (9.8)	NA	
				CH	43 (97.7)	1 (2.3)	NA	
				PGC	4 (100)	0 (0)	NA	
Do you regularly decontaminate the room after intubation in COVID-19 cases in your setup?	96 (42.5)	70 (31.0)	60 (26.5%)	TH/INI	48 (35)	47 (34.3)	42 (30.7)	0.06
				SGH	24 (58.5)	7 (17.1)	10 (24.4)	
				CH	23 (52.3)	14 (31.8)	7 (15.9)	
				PGC	1 (25)	2 (50)	1 (25.0)	

Intubation practice in COVID-19

Most of the responders said that intubation in COVID-19 patients was predominantly conducted by a senior anesthesiologist/intensivist (SA/IN; 37.2%) in the team and a senior resident (SR; 37.2%). When asked about the number of personnel present during intubation, 120 (53.1%) responders opted for two. Out of 120 responders, 71 (59.2%) were from TH/INI, followed by CH and SGH (21.7% vs. 17.5%; Table 3). Based on their experience, 41.7% of responders with one to five years of experience and 34.2% of responders with >10 years of experience confirmed the presence of two personnel during intubation.

**Table 3:** Induction and intubation protocols among different healthcare setups and their comparison. AT, anesthesia technical assistants; CC, circle system/closed circuit; CH, corporate hospitals; CS, clear sheets over the patients; CSS, closed suction system; DL, direct laryngoscope; ETT, endotracheal tube; FH, full hood without powered air purifier respirator; FH-PAPR, full hood with powered air purifier respirator; FOB, fiber-optic video bronchoscope/laryngoscope; GS, GlideScope; HFNO, high-flow nasal oxygenation; HMEF, heat and moisture exchanger and filter; IVF, intravenous fluid; MRSI, modified rapid sequence intubation; PG, junior resident/postgraduate/diploma; OSS, open suction system; PGC, peripheral government COVID-19 centers; RI, routine induction with intermediate acting muscle relaxants; RSI, rapid sequence intubation; RU, reusable equipment; SA/IN, senior anesthesiologist/intensivist in the team; SGH, state government hospital; SO, with sedation only; SR, senior resident; SU, single-use equipment; TH/INI, tertiary care hospital/institute of national importance; TIB, transparent intubation box; VI, visual inspection of chest rise and fumes in ETT; VL-IS, video laryngoscope with a built-in screen; VL-SS, video laryngoscope with a separate screen

Questionnaire	Options	Cumulative response, *n* (%)	Healthcare setup				*P*-value
			TH/INI, *n* (%)	SGH, *n* (%)	CH, *n* (%)	PGC, *n* (%)	
Who is conducting intubation in COVID-19 patients most of the time at your setup?	SA/IN	84 (37.2)	41 (48.8)	17 (20.2)	24 (28.6)	2 (2.4)	0.040
	SR	84 (37.2)	56 (66.7)	12 (14.3)	14 (16.7)	2 (2.4)	
	PG	58 (25.6)	40 (69)	12 (20.7)	6 (10.3)	0 (0)	
	AT	0	0	0	0	0	
How many personnel are present during intubation in your setup?	1	11 (4.9)	5 (45.5)	4 (36.4)	1 (9.1)	1 (9.1)	0.16
	2	120 (53.1)	71 (59.2)	21 (17.5)	26 (21.7)	2 (1.7)	
	3	80 (35.4)	54 (67.5)	14 (17.5)	11 (13.8)	1 (1.3)	
	>3	15 (6.6)	7 (46.7)	2 (13.3)	6 (40)	0 (0)	
Which kind of intubation equipment/kit do you use in your setup?	SU	51 (22.6)	27 (52.9)	14 (27.5)	8 (15.7)	2 (3.9)	0.29
	RU	100 (44.2)	63 (63)	16 (16)	19 (19)	2 (2)	
	Both	75 (33.2)	47 (62.7)	11 (14.7)	17 (22.7)	0 (0)	
What is the method of induction you practice for intubation in your setup?	RI	33 (14.6)	18 (54.5)	7 (21.2)	7 (21.2)	1 (3)	0.83
	RSI	105 (46.5)	64 (61)	22 (21)	17 (16.2)	2 (1.9)	
	MRSI	76 (33.6)	49 (64.5)	9 (11.8)	17 (22.4)	1 (1.3)	
	SO	12 (5.3)	6 (50)	3 (25)	3 (25)	0 (0)	
What are your considerations for postinduction hypotension?	Prophylactic IVF	67 (29.6)	38 (56.7)	13 (19.4)	14 (20.9)	2 (3)	0.38
	Post induction IVF	35 (15.5)	26 (74.3)	4 (11.4)	5 (14.3)	0 (0)	
	Prophylactic vasopressor	19 (8.4)	9 (47.4)	5 (26.3)	5 (26.3)	0 (0)	
	Post-induction vasopressor	20 (8.8)	12 (60)	7 (35)	1 (5)	0 (0)	
	Post-induction IVF and vasopressor	85 (37.6)	52 (61.2)	12 (14.1)	19 (22.4)	2 (2.4)	
What is your preferred induction agent while intubating COVID-19 cases?	Propofol	147 (65)	90 (61.2)	28 (19)	25 (17)	4 (2.7)	0.57
	Thiopentone	5 (2.2)	3 (60)	2 (40)	0 (0)	0 (0)	
	Ketamine	21 (9.3)	14 (66.7)	4 (19)	3 (14.3)	0 (0)	
	Etomidate	26 (11.5)	16 (61.5)	2 (7.7)	8 (30.8)	0 (0)	
	Midazolam	27 (11.9)	14 (51.9)	5 (18.5)	8 (29.6)	0 (0)	
What equipment do you use the most for intubating COVID-19 patients in your setup?	VL-SS	22 (9.7)	15 (68.2)	2 (9.1)	4 (18.2)	1 (4.5)	0.32
	VL-IS	55 (24.3)	41 (74.5)	8 (14.5)	6 (10.9)	0 (0)	
	GS	5 (2.2)	3 (60)	2 (40)	0 (0)	0 (0)	
	DL	142 (62.8)	77 (54.2)	29 (20.4)	33 (23.2)	3 (2.1)	
	FOB	2 (0.9)	1 (50)	0 (0)	1 (50)	0 (0)	
How do you prevent aerosol contamination during intubation?	TIB	84 (37.2)	49 (58.3)	17 (20.2)	18 (21.4)	0 (0)	0.03
	CS	104 (46)	64 (61.5)	16 (15.4)	23 (22.1)	1 (1)	
	Other	38 (16.8)	24 (63.2)	8 (21.1)	3 (7.9)	3 (7.9)	
What do you use to protect your face during intubation in COVID-19 cases in your setup?	Face shield	139 (61.5)	85 (61.2)	29 (20.9)	23 (16.5)	2 (1.4)	0.001
	Goggles	34 (15)	28 (82.4)	2 (5.9)	2 (5.9)	2 (5.9)	
	FH	41 (18.1)	21 (51.2)	8 (19.5)	12 (29.3)	0 (0)	
	FH-PAPR	12 (5.3)	3 (25)	2 (16.7)	7 (58.3)	0 (0)	
When do you attach the HMEF to ETT?	Before intubation	74 (32.7)	46 (62.2)	15 (20.3)	12 (16.2)	1 (1.4)	0.58
	After intubation	45 (19.9)	23 (51.1)	9 (20)	13 (28.9)	0 (0)	
	Just before connecting to the ventilator circuit	79 (35)	50 (63.3)	13 (16.5)	13 (16.5)	3 (3.8)	
	Time of attachment is variable	28 (12.4)	18 (64.3)	4 (14.3)	6 (21.4)	0 (0)	
What do you use for suctioning ETT in COVID-19 patients in your setup?	CSS	173 (76.5)	99 (57.2)	33 (19.1)	38 (22)	3 (1.7)	0.25
	OSS	53 (23.5)	38 (71.7)	8 (15.1)	6 (11.3)	1 (1.9)	

RSI and MRSI were practiced in most of the responder's hospitals (46.5% vs. 33.6%). Most responders were from TH/INI who chose RSI, followed by SGH (21%) and CH (16.2%). Responders with one to five years and >10 years of experience opted for RSI as the sole induction method (35.2% each). Most responders opted for propofol as the agent of choice for intubation across all healthcare setups. Postinduction hypotension was managed predominantly with intravenous fluid (IVF) and vasopressors after induction by 85 (37.6%) responders. In most centers, responders used direct laryngoscope (62.8%), whereas video laryngoscope (VL) was used by 34% of responders. Using reusable equipment for intubation was chosen by 100 (44.2%) responders, of which 63% were from TH/INI.

A total of 104 (46%) responders used clear sheets (CSs) for the prevention of aerosol contamination during intubation, and a transparent intubation box (TIB) was used by 37.2% of responders. Compared to others, 61.5% of responders from TH/INI used CS, which was statistically significant (Table 3). The use of a face shield was the most common method of protecting the face from contamination (61.5%), and the distribution among the healthcare setup was statistically significant (*P* < 0.05; Table 3). Thirty-five percent of responders used to attach the HMEF before connecting to the ventilator circuit, whereas 74 (32.7%) responders chose to attach HMEF before intubation (Table 3). Most of the responders said that they used CSS (76.5%) for suctioning at their center; responders with >10 years of experience predominantly selected CSS (39.9%) over other responders, which was statistically significant (*P* < 0.05; Table 4).

**Table 4:** Induction and intubation protocols according to the experience level and their comparison. CC, circle system/closed circuit; CH, corporate hospital; CS, clear sheet over the patient; CSS, closed suction system; DL, direct laryngoscope; ETT, endotracheal tube; FH, full hood without powered air purifier respirator; FH-PAPR, full hood with powered air purifier respirator; FOB, fiber-optic video bronchoscope/laryngoscope; GS, GlideScope; HMEF, heat and moisture exchanger and filter; IVF, intravenous fluid; MRSI, modified rapid sequence intubation; OSS, open suction system; RI, routine induction with intermediate-acting muscle relaxants; RSI, rapid sequence intubation; RU, reusable equipment; SU, single-use equipment; TIB, transparent intubation box; VI, visual inspection of chest rise and fumes in ETT; VL-IS, video laryngoscope with a built-in screen; VL-SS, video laryngoscope with a separate screen

Questions		Cumulative response, *n* (%)	Year of experience (in years)				*P*-value
			<1, *n* (%)	1-5, *n* (%)	5-10, *n* (%)	>10, *n* (%)	
How many personnel are present during intubation in your setup?	1	11 (4.9)	0 (0)	5 (45.5)	3 (27.3)	3 (27.3)	0.15
	2	120 (53.1)	8 (6.7)	50 (41.7)	21 (17.5)	41 (34.2)	
	3	80 (35.4)	5 (6.3)	20 (25)	25 (31.3)	30 (37.5)	
	>3	15 (6.6)	3 (20)	4 (26.7)	3 (20)	5 (33.3)	
Which kind of intubation equipment/kit do you use in your setup?	SU	51 (22.6)	3 (5.9)	19 (37.3)	9 (17.6)	20 (39.2)	0.002
	RU	100 (44.2)	9 (9)	40 (40)	31 (31)	20 (20)	
	Both	75 (33.2)	4 (5.3)	20 (26.7)	12 (16)	39 (52)	
What is the method of induction you practice for intubation in your setup?	RI	33 (14.6)	4 (12.1)	13 (39.4)	5 (15.2)	11 (33.3)	0.37
	RSI	105 (46.5)	5 (4.8)	37 (35.2)	26 (24.8)	37 (35.2)	
	MRSI	76 (33.6)	7 (9.2)	25 (32.9)	18 (23.7)	26 (34.2)	
	SO	12 (5.3)	0 (0)	4 (33.3)	3 (25)	5 (41.7)	
What are your considerations for postinduction hypotension?	Prophylactic IVF	67 (29.6)	4 (6)	22 (32.8)	17 (25.4)	24 (35.8)	0.98
	Post induction IVF	35 (15.5)	4 (11.4)	10 (28.6)	10 (28.6)	11 (31.4)	
	Prophylactic vasopressor	19 (8.4)	2 (10.5)	6 (31.6)	4 (21.1)	7 (36.8)	
	Postinduction vasopressor	20 (8.8)	1 (5)	8 (40)	4 (20)	7 (35)	
	Postinduction IVF and vasopressor	85 (37.6 )	5 (5.9)	33 (38.8)	17 (20)	30 (35.3)	
What is your preferred induction agent while intubating COVID-19 cases?	Propofol	147 (65)	13 (8.8)	49 (33.3)	34 (23.1)	51 (34.7)	0.59
	Thiopentone	5 (2.2)	0 (0)	3 (60)	1 (20)	1 (20)	
	Ketamine	21 (9.3)	1 (4.8)	9 (42.9)	3 (14.3)	8 (38.1)	
	Etomidate	26 (11.5)	0 (0)	12 (46.2)	7 (26.9)	7 (26.9)	
	Midazolam	27 (11.9)	2 (7.4)	6 (22.2)	7 (25.9)	12 (44.4)	
What equipment do you use most for intubating COVID-19 patients in your setup?	VL-SS	22 (9.73)	0 (0)	8 (36.4)	6 (27.3)	8 (36.4)	0.19
	VL-IS	56 (24.78)	3 (5.36)	16 (28.57)	7 (12.50)	30 (53.57)	
	GS	5 (2.21)	0 (0)	2 (40)	1 (20)	2 (40)	
	DL	142 (62.83)	13 (9.2)	53 (37.3)	37 (26.1)	39 (27.5)	
	FOB	1 (0.04)	0	0	0	1 (100)	
How do you prevent aerosol contamination during intubation?	TIB	84 (37.2)	5 (6)	31 (36.9)	20 (23.8)	28 (33.3)	0.74
	CS	104 (46)	7 (6.7)	32 (30.8)	24 (23.1)	41 (39.4)	
	Other	38 (16.8)	4 (10.5)	16 (42.1)	8 (21.1)	10 (26.3)	
What do you use to protect your face during intubation in COVID-19 cases in your setup?	Face shield	139 (61.5)	7 (5)	52 (37.4)	29 (20.9)	51 (36.7)	0.38
	Goggles	34 (15)	4 (11.8)	12 (35.3)	9 (26.5)	9 (26.5)	
	FH	41 (18.1)	4 (9.8)	13 (31.7)	8 (19.5)	16 (39)	
	FH-PAPR	12 (5.3)	1 (8.3)	2 (16.7)	6 (50)	3 (25)	
When do you attach the HMEF to ETT?	Before intubation	7 (32.7)	4 (5.4)	24 (32.4)	18 (24.3)	28 (37.8)	0.83
	After intubation	45 (19.9)	3 (6.7)	18 (40)	7 (15.6)	17 (37.8)	
	Just before connecting to the ventilator circuit	79 (35)	5 (6.3)	28 (35.4)	20 (25.3)	26 (32.9)	
	Time of attachment is variable	28 (12.4)	4 (14.3)	9 (32.1)	7 (25)	8 (28.6)	
What do you use for suctioning ETT in COVID-19 patients in your setup?	CSS	173 (76.5)	14 (8.1)	54 (31.2)	36 (20.8)	69 (39.9)	0.01
	OSS	53 (23.5)	2 (3.8)	25 (47.2)	16 (30.2)	10 (18.9)	

We have got varied responses to the multiple-answer questions from the responders. We calculated each option individually and represented their response as percentages. Intubation of COVID-19 patients was considered when they failed on noninvasive ventilation (NIV) and high-flow nasal oxygenation (HFNO) by the majority of the responders (96.02%) across all healthcare setups. Cardiopulmonary arrest was the second most common indication for intubation as per the responders (58.85%). The most common method of oxygenation and ventilation strategy was via a facemask with reservoir (FMR; 55.8%) followed by HFNO (32.3%). Most responders opted for BM (41.1%) for preoxygenation over CC (25.2%). Responders favored second-generation supraglottic airway devices (SGA2; 57.5%) and classic laryngeal mask airway (C-LMA; 56.1%) as rescue devices in case of a difficult airway (Table 5). When asked about their confirmation method for an appropriate ETT position, 66.3% of responders said visual inspection followed by end-tidal carbon dioxide waveform (EtCO2) tracing (53.9%).

**Table 5:** Indication and preparation for intubation among different healthcare setups and their comparison. BM, bag and mask; CC, circle system/closed circuit; CH, corporate hospital; C-LMA, classic laryngeal mask airway; EtCO2, end-tidal carbon dioxide waveform; ETT, endotracheal tube; FMR, face mask with the reservoir; HFNO, high-flow nasal oxygenation; NIV, noninvasive ventilation; PGC, peripheral government COVID-19 centers; SGA2, second-generation supraglottic airway device; SGH, state government hospitals; TH/INI, tertiary care hospital/institute of national importance; VI, visual inspection of chest rise and fumes in ETT

Questionnaires	Options	Cumulative response, *n* (%)	Healthcare setup, *n* (%)				*P*-value
			TH/INI	SGH	CH	PGC	
When do you consider intubation in COVID-19 patients?	Failed noninvasive ventilation/high-flow nasal oxygenation	217 (96.02)	132 (60.83)	37 (17.05)	44 (20.28)	4 (1.84)	0.03
	Cardiopulmonary arrest	133 (58.85)	84 (63.16)	23(17.29)	26 (19.55)	0 (0)	
What are the strategies that you consider for ventilation and oxygenation before choosing intubation?	FMR	126 (55.8)	77 (61.1)	20 (15.9)	25 (19.8)	4 (3.2)	0.47
	HFNO	73 (32.3)	44 (60.3)	13 (17.8)	16 (21.9)	0 (0)	
What is the method of preoxygenation that you use in your setup?	BM	93 (41.15)	56 (60.22)	21 (22.58)	15 (16.13)	1 (2.33)	0.06
	CC	57 (25.22)	27 (47.37)	16 (28.07)	14 (24.56)	0 (0)	
What rescue airway devices do you keep in the intubation kit/trolley?	SGA2	130 (57.52)	80 (61.54)	20 (15.38)	26 (20)	4 (3.08)	0.41
	C-LMA	127 (56.19)	76 (59.84)	23 (18.11)	27 (21.26)	1 (0.79)	
How do you confirm the correct position of ETT in COVID-19-intubated patients in your setup?	VI	150 (66.3)	95 (63.33)	25 (16.67)	27 (18)	3 (2)	0.00
	EtCO2	122 (54)	75 (61.48)	19 (15.57)	27 (22.13)	1 (0.82)	

## Discussion

COVID-19 is a disease under evaluation involving multiple systems, predominantly affecting the respiratory system. Although pneumonia is the most severe manifestation, sometimes it ends up with acute respiratory distress syndrome (ARDS) requiring some form of mechanical ventilation [5]. A recent study on a larger population in the United Kingdom (excluding Scotland) showed that about 36.9% were affected by severe ARDS during their critical care unit and 72% received invasive ventilation [6]. This survey has been aimed at knowing current practices in airway management and their aptness as per the recent guidelines.

Planning

Teaching and Training

Airway management, in particular, is considered the highest AGP, which needs appropriate simulation and training before proceeding with airway care during COVID-19 [7]. Our study results showed an overall poor trend toward training. Sixty-three percent of responders did not receive training, and the least was found in corporate hospitals (70.5%) compared to other setups. It is suggested to conduct airway simulation and team management training for a good outcome during AGPs.

Personal Protective Equipment (PPE)

Cook emphasized the following sequence of donning and doffing of PPE to decrease aerosol contamination [8]. Donning and doffing of PPE were mainly followed as per ICMR guidelines in all setups, speaking of awareness uniformity. Cook also suggested using goggles or visors and FFP3 masks for facial protection during AGP [8]. Additionally, patients' use of a fluid-resistant surgical mask gives further protection. Our survey noticed the use of face shields in abundance. Face shield respirators or powered air-purifying respirators (PAPRs) can be used additionally but are not mandatory [8]. This survey also showed limited use of PAPRs in our country.

Intubation Area Preparedness

Negative-air pressurized rooms are ideal for preventing aerosol contamination. Indian Society of Critical Care Medicine (ISCCM) for airway management in COVID-19 patients also recommended the same [9]. Our study found a need for preparedness in various healthcare setups for such facilities. Introspection of hospital management is required to limit in-hospital transmission of COVID-19.

A heat moisture exchanger and filter (HMEF) is necessary to humidify and filtrate viral particles. It is recommended to attach two HMEFs between the patient and the machine end at the commencement of preoxygenation - one between the facemask and circuit and another between the expiratory limb and ventilator to lessen the burden of viral load [4,8]. Most responders used to attach the HMEF just before connecting the patient to the ventilator. Compliance among the senior responders was comparatively more.

Another AGP of concern is suctioning of ETT, which urges the use of a closed suction system. Imbriaco and Monesi have emphasized considering it mandatory to use CSS for patients in the ICU with an artificial airway to reduce bioaerosol exposure [10]. Impressively, most responders in their centers use CSS for suctioning.

Resource Availability

Single-use equipment is better than reusable one for infection control. A disposable fiberscope is preferred for awake tracheal intubation [4]. However, the cost of intubation for disposable was high [11]. Corporate hospitals used disposable and reusable equipment equally, whereas tertiary care hospitals use reusable ones more often, per the survey. As TH caters to a larger COVID-19 population, it is most economical to utilize reusable items while keeping the safety of the frontline workers in mind. Second-generation supraglottic airway devices (SGA2) and classic laryngeal mask airways (C-LMAs) were chosen by maximum responders as rescue measures in difficult airway situations. SGA2 is the safest device for aerosol generation and facilitates bronchoscopic asleep intubation while maintaining oxygenation [10,12,13]. Participants from TH/INI, SGH, and CH have chosen both devices equally. We suggest the use of SGA2 over C-LMA to curtail transmission risk.

Team Preparation

During high AGPs like intubation, it is always advisable to limit personnel in the procedure area. The AIDAA has recommended a minimum of two personnel with appropriate skills during the procedure [4]. Most of the responders across all healthcare setups managed intubation with two personnel. Taking the risk into account, Cheung et al. recommended that intubation should be done by experts [14]. Our survey found that SAs/INs and SRs in various setups conducted intubation.

Additional Safety Equipment

Various methods have been adopted to decrease aerosol exposure during intubation, such as aerosol boxes, transparent sheets, and plastic tents [15]. However, there is no recommendation found in support of such measures. Instead, unconventional devices cause restriction of hand movements and possible difficulty in visualization [4]. The survey showed a majority of setups use CSs during intubation. We suggest the use of familiar instruments during laryngoscopy.

Alternate Oxygenation and Ventilation Strategies

COVID-19 patients with hypoxemic respiratory failure were recommended for high-flow nasal cannula (HFNC) oxygen over noninvasive positive pressure ventilation (NIPPV) by the National Institute of Health (NIH) if the conventional oxygen therapy failed [16]. Our survey's most common oxygenation mode was an FMR, followed by HFNC. 

Indication for Intubation

Per our survey, NIV/HFNC failure was a significant reason for intubation. Other indications were cardiopulmonary arrest, altered mental status, and acute hypoxemia. In their review, Pisano et al. suggested that the PaO_2_/FiO_2_ ratio and chest computed tomography findings are insufficient for predicting tracheal intubation [17]. The most common indications for intubation are altered mental status, increased work of breathing, severe hypoxemia not responding to NIPPV, and severe acidosis [18,19]. Literature has described the importance of early recognition of NIPPV failure for escalating therapy to invasive ventilation to reduce morbidity and mortality, and the *respiratory rate oxygenation* (ROX) index and mortified ROX index in patients with pneumonia with acute respiratory failure treated with HFNO have been reported and validated to identify such patients [19,20]. Most healthcare facilities do not use any scoring system for the same - a severe fallout of ICUs in India. Using a scoring system more often objectifies the cause and avoids a catastrophe.

Airway management

Preoxygenation

As per recommendations by the AIDAA, preoxygenation should be done with an appropriate-size face mask and a two-hand technique with tidal volume breathing using a closed circuit. Further, the association advised against using HFNO and NIV for preoxygenation to avoid aerosol generation. Contrary to the above, the maximum number of participants across all healthcare setups used bag masks in our survey. Training and simulation should be more toward AGPs during intubation [4].

Induction and Intubation

In our survey, the preferred induction agent during intubation was propofol, unanimously across all the healthcare setups and the participants of different experience levels. Ketamine was chosen for induction in sepsis or septic shock, as it is protective against inflammation, reduces nitric oxide production, and decreases cardiac dysfunction, all of which prevent hemodynamic instability [21]. In contrast, propofol is known to cause postinduction hypotension and more so in sepsis because of further reduction in cardiac inotropy and lusitropy by approximately 40%. It is advisable to choose ketamine over propofol in severe sepsis cases of COVID-19 [21,22]. RSI curtails the induction time and avoids mask ventilation, ultimately reducing aerosol generation [23]. Consultants with more than 10 years and one to five years of experience preferred RSI over routine intubation. The newly joined residents with less than one year of experience had mixed thoughts, which can improve with training and simulation.

Most of the guidelines suggested the use of a VL over direct laryngoscopy. It has been shown to improve the laryngeal view and first-pass success rate and lower the incidence of the need for external laryngeal manipulation [24]. Our survey revealed the predominant use of direct laryngoscopy in various setups. Lack of availability and cost were significant barriers to using a VL.

Several setups adopt ETT clamping during intubation to prevent aerosol contamination [9,25]. According to our survey, in India, surprisingly, it was practiced by only 39%. Healthcare workers across different setups must update themselves about recent guidelines for intubation. It is suggested to manage postinduction hypotension with phenylephrine at a dose of 10-20 microg administered, preferably just before induction. Unmonitored IVF administration in the presence of acute lung injury may be detrimental. Conservative fluid administration and transient vasopressor use may be helpful [22,26]. Most participants preferred IVF and vasopressor in the postinduction phase in this survey. Judicious fluid and vasopressor administration in ARDS improves prognosis [27].​​​​​​​

Confirmation of Intubation

Most of the guidelines, including the AIDAA, recommended the use of EtCO2 for confirmation of the correct position of ETT after intubation. To our surprise, most participants opted for visual inspection instead of EtCO2 tracing. We suggest using ultrasound to confirm tube position along with EtCO2 trace. Further studies will be required supporting ultrasound use in COVID-19 intubations [4,28].

Postintubation Decontamination Policy

Decontamination of the procedure room was followed by <50% of the responder's hospital. State government and corporate hospitals followed disinfection policies actively compared to the tertiary care hospitals as per the survey result. The reason may be attributed to the caseload taken by tertiary care hospitals. As per the Ministry of Health and Family Welfare (MoHFW) directives in the initial phase, COVID-19 patients were mainly confined to tertiary care hospitals. Lack of workforce and institutional policy in the face of a new pandemic may contribute to policy failure [4].

In an open-ended question, we asked for factors creating hindrance to intubation; 64% found no such factor preventing them from intubation. About 16% cited the nonavailability of PPE as a cause, and 11% stated their unwillingness to intubate. Physicians still need to understand about pathophysiology and prevention strategies of COVID-19, which may answer the refusal.

Our study had a few limitations. First, it covered only some aspects of airway management. Second, most responders are confined to tertiary care centers. Third, as COVID-19 is in a dynamic phase, the suggestions made in this study might change over a while.

## Conclusions

Most of the responders were following safe intubation practices as suggested by the AIDAA. However, critical aspects, which might seriously challenge safety, such as preoxygenation practice; equipment for intubation such as using VLs over direct laryngoscopy; and method of confirmation of ETT using EtCO2 over direct visualization still have scopes for further improvements. Healthcare facilities may formulate policies best suited for their setups based on the available resources and in line with the guidelines. As the evidence and technologies/innovations evolve and get validated in prospective studies, all physicians should keep updating themselves with newer airway management strategies.
